# Supplementary material for: A label-free optical biosensor-based point-of-care test for the rapid detection of Monkeypox virus
Source: Biosens Bioelectron. Author manuscript; Available in PMC 2026 Mar 5. (PMC12962341; doi:10.1016/j.bios.2024.116932)
Supplement: Supplementary material [file NIHMS2144903-supplement-Supplementary_material.pdf]

A Label-free Optical Biosensor-Based Point-of-Care Test for the Rapid Detection of Monkeypox Virus

Mete Aslan[1], Elif Seymour[2], Howard Brickner[3], Alex E. Clark[3], Iris Celebi[1], Michael B. Townsend[4], Panayampalli S. Satheshkumar [4], Megan Riley[5], Aaron F. Carlin[3,6], M. Selim Ünlü[1,7]*, and Partha Ray[3]*

[1.] Department of Electrical and Computer Engineering, Boston University, Boston, MA, 02215, USA

[2.]iRiS Kinetics, Boston University, Business Incubation Center, Boston, MA, 02215, USA

[3.] Department of Medicine, Division of Infectious Diseases and Global Public Health, University of California, San Diego, CA 92093, USA

[4.] Poxvirus and Rabies Branch, Centers for Disease Control and Prevention, Atlanta, GA 30329, USA

[5.]axiVEND, Winter Garden, FL 34787, USA

[6.] Department of Pathology, University of California, San Diego, CA 92093, USA

[7.] Department of Biomedical Engineering, Boston University, Boston, MA, 02215, USA

*Address correspondence to:

Partha Ray (pray@health.ucsd.edu), M. Selim Ünlü (selim@bu.edu)

Confidential

Supplementary Materials:

MPXV A29 protein ELISA: For ELISA with the MPXV A29 protein, the lyophilized A29 recombinant protein from strain *MPXV-ZAI-96-I-16 (Clade I) was purchased (Arco Biosystems, Catalogue number* A2L-M52H3-100 μg) and dissolved in sterile water to make a stock of 400 μg/mL. Next, A29 protein dilutions at the indicated concentrations were made in the carbonate-bicarbonate (pH 9.4) buffer (ThermoFisher Scientific Catalogue: 28382), and 100 µL of the diluted solutions were added to each well. All the subsequent steps followed were the same as described in the *Materials and Methods* for the inactivated viruses. All the assays were conducted in triplicate (n=3) sets at every concentration for statistical significance and Limit of Detection (LOD) calculations. The threshold signal is calculated as an average signal from the negative control BSA plus three standard deviations. LOD is calculated as the concentration value corresponding to the point where the dilution curve intersects the threshold line.

Mpox quantitative PCR (qPCR): The genomic DNA from 100 µl heat-inactivated mpox samples was extracted in 50 µl buffer using the Zymo DNA miniprep kit (Catalog: D3024 Zymo Research, Irvine, CA) following the manufacturer's instructions. 4 µl of this DNA sample at indicated dilutions were used to run the assay. For the mpox quantitative PCR (qPCR), we used primers: F3L forward 5´-CATCTATTATAGCATCAGCATCAGA-3´ and reverse 5´-GATACTCCTCCTCGTTGGTCTAC -3´, which annealed once per genome (Maksyutov et al., 2016; Mills et al., 2023). qPCR was performed on a QuantStudio5 qPCR machine (Thermo Fisher Scientific, Waltham, MA) using iTaq Universal SYBR Green Supermix (Catalog: 1725121 Bio-Rad, Hercules, California) in 10µl reactions. A standard curve included known copy numbers (range 10^4-10^9) of purified 499bp fragments containing the qPCR amplicon. The fragment was amplified from the same viral DNA extraction using Q5® High-Fidelity 2X Master Mix (catalog: M0492S, New England Biolabs, Ipswich, MA) with primer pair 1-F 5′-ACAGGGTTAACACCTTTCCAATA-3′ + 1-R 5′-AATCTCCAGAACCAGCATCAC-3′ (Clark et al., 2023). The product was visualized as a single band on agarose gel, purified with a DNA Clean and Concentrate kit (catalog D4014, Zymo Research, Irvine, CA), and quantified on a NanoPhotometer P360 (Implen, Munich, Germany). SYBR Green melt curve indicated one size of amplicon in all qPCR samples.

Confidential

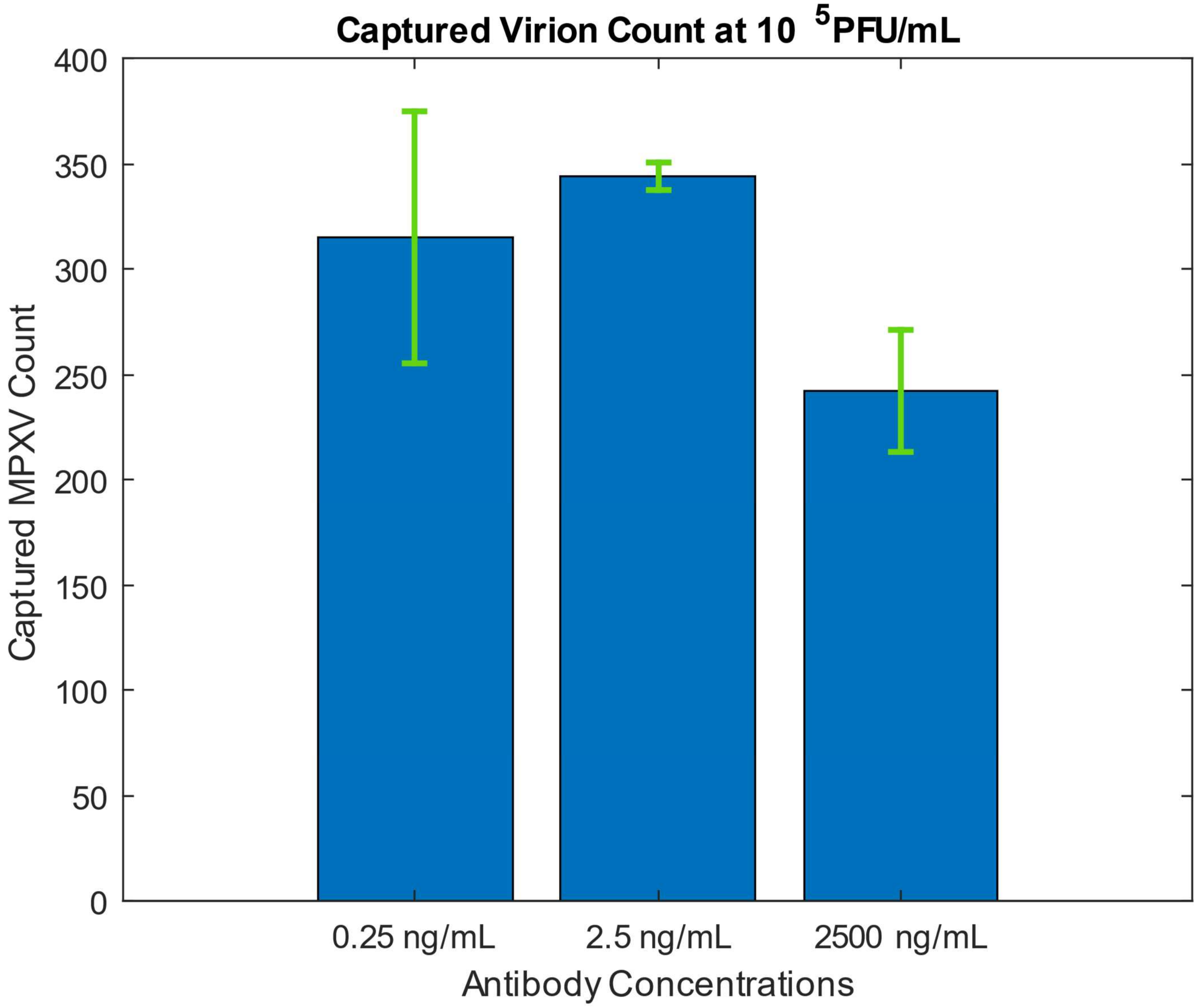

Supplementary Figure 1: Captured particle counts of different homogenous assays with varying anti-A29 antibody concentrations. The spot size is the same for all measurements. Three different protein G spots are analyzed for each concentration. The bar graphs represent triplicate sample (n=3) measurements' Mean +/- Standard Deviation (error bars).

Confidential

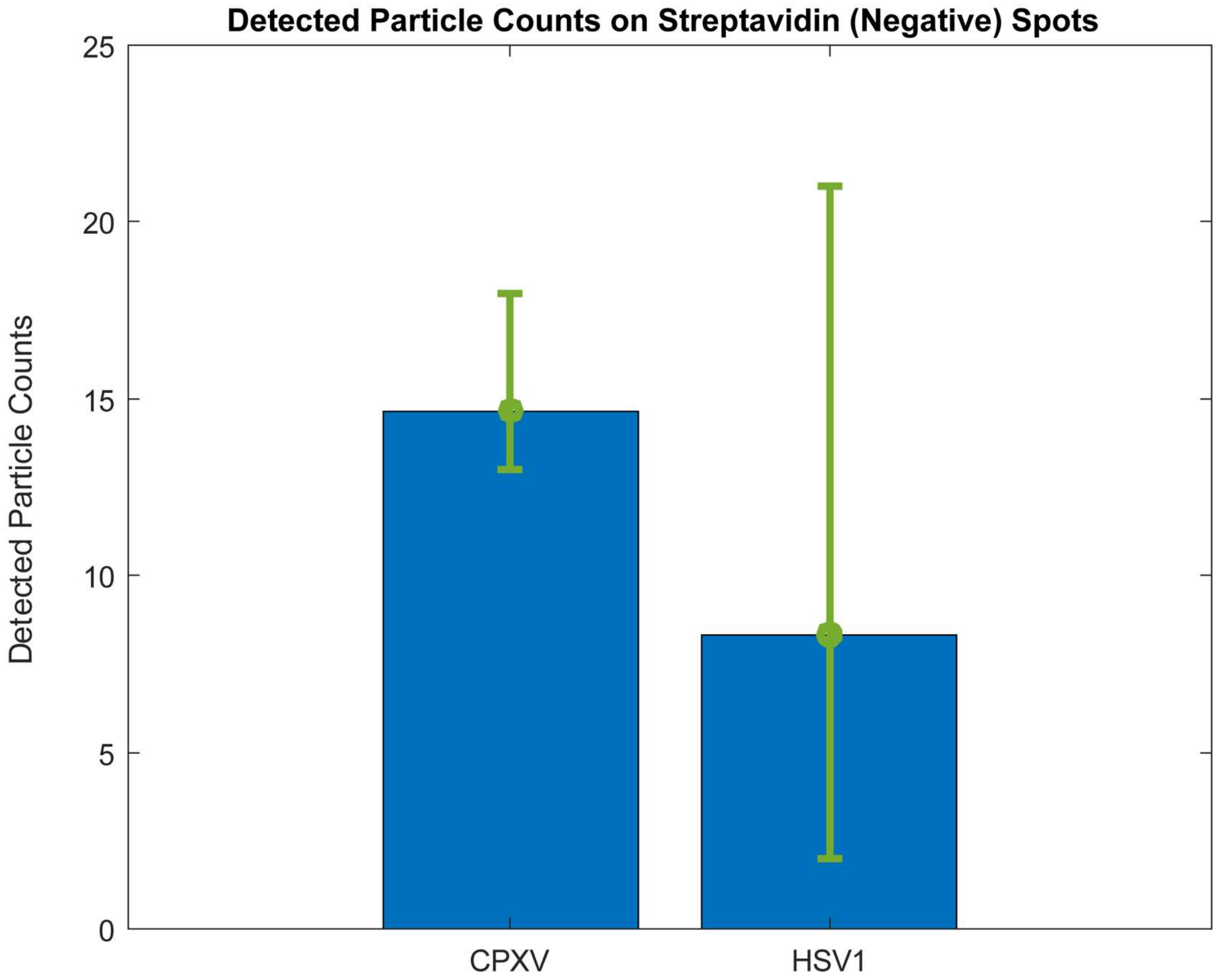

Supplementary Figure 2: Detected particle counts on negative control spots for specificity experiments. The spot size is the same for all measurements. Three different streptavidin spots are analyzed for each virus incubation. The bar graphs represent triplicate sample (n=3) measurements' Mean +/- Standard Deviation (error bars).

Confidential

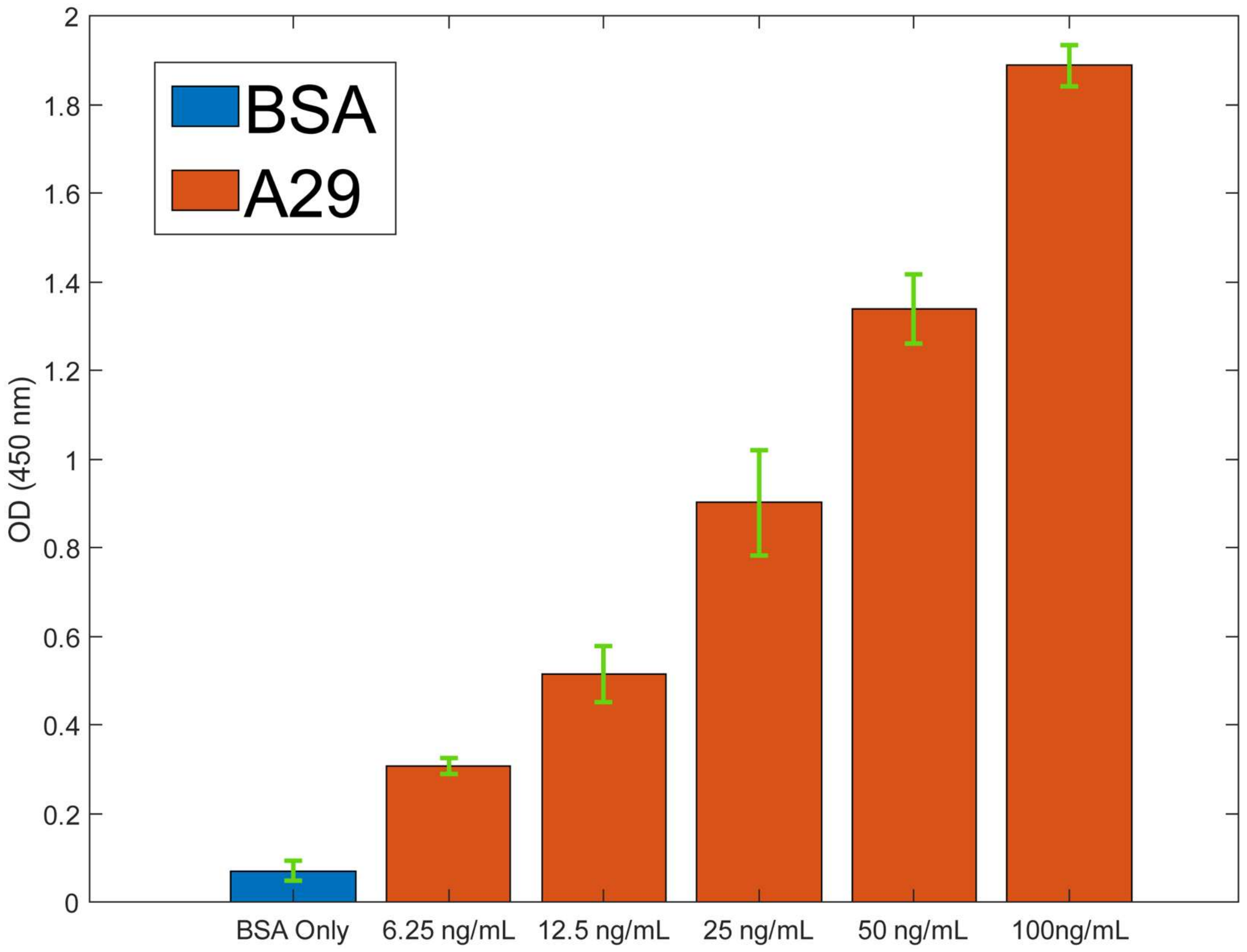

Supplementary Figure 3: Results of ELISA experiment to validate the Mpox mAb (mAb 69-126-3) using (Hughes et al., 2014) MPXV A29 protein from strain *MPXV-ZAI-96-I-16 (Clade I)*. The bar graphs show triplicate sample (n=3) measurements' Mean with error bars representing Standard Deviation.

Confidential

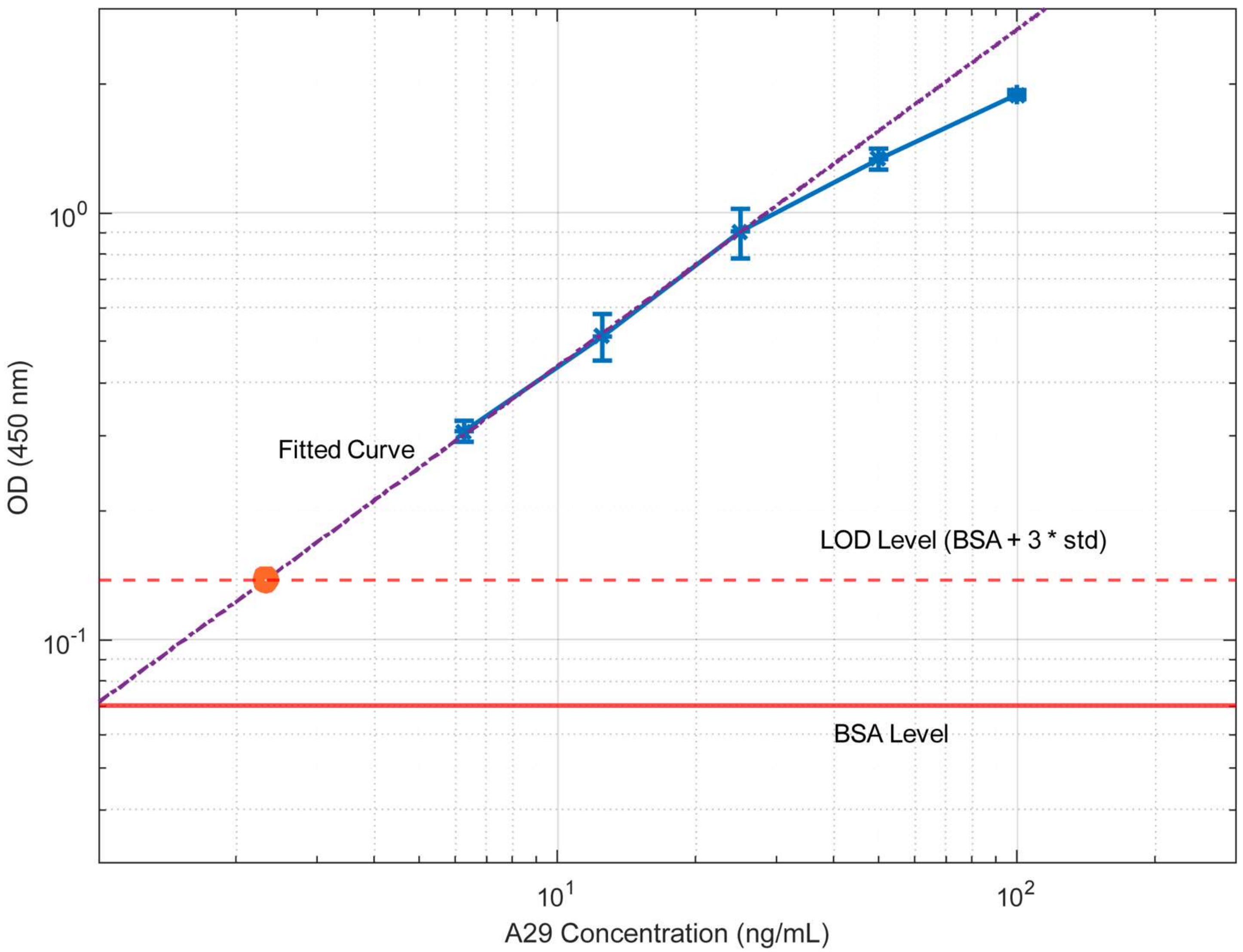

Supplementary Figure 4: ELISA LOD curve for A29 protein antigen detection using Mpox mAb 69-126-3. The graph is the mean +/− SD of OD450 values from triplicate samples. The best-fit line was calculated using log-log transformed values. The calculated LOD is ~2.32 ng/mL.

Confidential

Antibody / Virus particle Ratio at 10^5 PFU/mL	Antibody Concentration (ng/mL)
~8300000:1	2500
~8300:1	2.5
~830:1	0.25

Supplementary Table 1: Antibody to Virus particle ratio calculations. Given that the molecular weight of the anti-A29 antibody is 181 kDa and the viral particle-to-PFU ratio is ~10, the number of antibody molecules in a homogenous assay is calculated from its concentration in (ng/mL)

Confidential

	Quantity Mean	Quantity SD	Ct Mean	Ct SD	µl DNA in assay	serial dilution	additional dilution factor	copies/µl	copies/ml
Sample 1	7,359,631.50	357,962.19	12.48	0.08	4.00	1.00	0.50	919953.938	9.20E+08
Sample 2	955,357.94	15,385.41	15.69	0.03	4.00	10.00	0.50	1194197.42	1.19E+09
Sample 3	118,968.88	5,247.74	18.97	0.07	4.00	100.00	0.50	1487110.94	1.49E+09
Sample 4	12,224.17	908.72	22.55	0.12	4.00	1,000.00	0.50	1528021.73	1.53E+09
Sample 5	1,298.42	36.60	26.07	0.04	4.00	10,000.00	0.50	1623031.01	1.62E+09
Sample 6	164.65	75.27	29.40	0.75	4.00	100,000.00	0.50	2058139.04	2.06E+09

Supplementary Table 2: Estimating mpox genomic copies/mL using qPCR: Using qPCR methods, we calculated that the heat-inactivated mpox stock of 2.65x 10^7 PFU/ml is 1.19x10^9 genome copies/ml (from 1:10 dilution). Therefore, our calculated PD-IRIS LOD for detecting MPXV of 200 PFU/ml corresponds to 8.98x10^3 copies/ml. The mpox test sample quantities were calculated using a standard curve. The mean of duplicate sets (n=2) is reported.

Confidential

Target	LODs in Buffer (Molarity)	Time (mins)	Approaches	POC applicability	Refs.
A29	0.35 nM (5 ng/mL)	5	Surface Enhanced Raman Spectroscopy	No	(Zhang et al., 2023)
A29	43.06 pM (0.62 ng/mL)	14	Biolayer Interferometry	No	(Song et al., 2024)
**MPXV (Whole Virus)**	**3.3 aM**	**20**	**PD-IRIS**	**Yes**	**This work**

Supplementary Table 3: Comparator table of Mpox detection assays utilizing MPXV A29 antibodies.

Confidential

Supplementary Video: A movie and the dynamic graph created from acquired images demonstrating real-time MPXV binding to a Protein G (positive) and Streptavidin (negative) spot.

Confidential

REFERENCES:

Clark, A. E., Furst, A., Sejane, K., Stellwagen, L., Proost, M., Pride, D., Smith, D. M., Carlin, A. F., & Bode, L. (2023). Validating Tools to Detect and Inactivate Monkeypox Virus in Human Milk. *Breastfeeding Medicine : The Official Journal of the Academy of Breastfeeding Medicine*, *18*(10), 785–789. https://doi.org/10.1089/BFM.2023.0175

Hughes, L. J., Goldstein, J., Pohl, J., Hooper, J. W., Lee Pitts, R., Townsend, M. B., Bagarozzi, D., Damon, I. K., & Karem, K. L. (2014). A highly specific monoclonal antibody against monkeypox virus detects the heparin binding domain of A27. *Virology*, *464–465*(1), 264–273. https://doi.org/10.1016/J.VIROL.2014.06.039

Maksyutov, R. A., Gavrilova, E. V., & Shchelkunov, S. N. (2016). Species-specific differentiation of variola, monkeypox, and varicella-zoster viruses by multiplex real-time PCR assay. *Journal of Virological Methods*, *236*, 215–220. https://doi.org/10.1016/J.JVIROMET.2016.07.024

Mills, M. G., Juergens, K. B., Gov, J. P., McCormick, C. J., Sampoleo, R., Kachikis, A., Amory, J. K., Fang, F. C., Pérez-Osorio, A. C., Lieberman, N. A. P., & Greninger, A. L. (2023). Evaluation and clinical validation of monkeypox (mpox) virus real-time PCR assays. *Journal of Clinical Virology : The Official Publication of the Pan American Society for Clinical Virology*, *159*. https://doi.org/10.1016/J.JCV.2022.105373
